# Ruminal and feces metabolites associated with feed efficiency, water intake and methane emission in Nelore bulls

**DOI:** 10.1038/s41598-023-45330-w

**Published:** 2023-10-21

**Authors:** Jessica Moraes Malheiros, Banny Silva Barbosa Correia, Caroline Ceribeli, Jennifer Jessica Bruscadin, Wellison J. S. Diniz, Priyanka Banerjee, Dielson da Silva Vieira, Tainã Figueiredo Cardoso, Bruno Gabriel Nascimento Andrade, Juliana Petrini, Daniel Rodrigues Cardoso, Luiz Alberto Colnago, Stanislau Bogusz Junior, Gerson Barreto Mourão, Luiz Lehmann Coutinho, Julio Cesar Pascale Palhares, Sergio Raposo de Medeiros, Alexandre Berndt, Luciana Correia de Almeida Regitano

**Affiliations:** 1 Embrapa Southeast Livestock, São Carlos, São Paulo Brazil; 2 Institute of Chemistry, University of São Paulo/USP, São Carlos, São Paulo Brazil; 3 Department of Food Science, University of Copenhagen, Copenhagen, Denmark; 4 Departament of Animal Sciences, Auburn University, Auburn, AL 36849 USA; 5 Computer Science Department, Munster Technological University, MTU/ADAPT, Cork, Ireland; 6 Department of Animal Science, University of São Paulo/ESALQ, Piracicaba, São Paulo Brazil; 7 Embrapa Instrumentation, São Carlos, São Paulo Brazil

**Keywords:** Genetics, Molecular biology, Environmental sciences

## Abstract

The objectives of this study were twofold: (1) to identify potential differences in the ruminal and fecal metabolite profiles of Nelore bulls under different nutritional interventions; and (2) to identify metabolites associated with cattle sustainability related-traits. We used different nutritional interventions in the feedlot: conventional (Conv; n = 26), and by-product (ByPr, n = 26). Thirty-eight ruminal fluid and 27 fecal metabolites were significantly different (*P* < 0.05) between the ByPr and Conv groups. Individual dry matter intake (DMI), residual feed intake (RFI), observed water intake (OWI), predicted water intake (WI), and residual water intake (RWI) phenotypes were lower (*P* < 0.05) in the Conv group, while the ByPr group exhibited lower methane emission (ME) (*P* < 0.05). Ruminal fluid dimethylamine was significantly associated (*P* < 0.05) with DMI, RFI, FE (feed efficiency), OWI and WI. Aspartate was associated (*P* < 0.05) with DMI, RFI, FE and WI. Fecal C22:1n9 was significantly associated with OWI and RWI (*P* < 0.05). Fatty acid C14:0 and hypoxanthine were significantly associated with DMI and RFI (*P* < 0.05). The results demonstrated that different nutritional interventions alter ruminal and fecal metabolites and provided new insights into the relationship of these metabolites with feed efficiency and water intake traits in Nelore bulls.

## Introduction

Feed accounts for the largest investments in beef cattle farming when the costs of acquisition of the animals is not considered^1–3^. Feed efficiency (FE) is directly associated with increased meat production per unit of feed intake. Higher FE can additionally contribute to achieving low greenhouse gas emissions^4^, as ruminants are considered the most critical enteric methane emitters^5^, directly affecting environmental footprint^6^.

Strategies aimed at increasing FE and mitigating methane emission (ME) comprise an important field of research to improve ruminal fermentation^7,8^. The rumen harbors a complex microbial ecosystem that converts plant biomasses to microbial proteins, volatile fatty acids and gases^9–11^. Thus, any change in the microbial population of this compartment can affect food degradation, nutrient availability, and, consequently, FE and ME^12,13^. However, mechanisms related to these phenotypes are complex and still not fully understood^14^.

The microbiome operates as an intermediate between diet and animal performance^15^. Different nutritional interventions can modify rumen microbiome characteristics^16–18^, affect food degradation and the availability of metabolites^12,19,20^. In this context, ruminal fluid metabolites are also promising for the prediction of FE and ME^13,16,21–24^.

The rumen and fecal microbiomes harbor structured populations whose cooccurrences may reflect their relationships^25–28^. A comparative approach using the experimental animals in the current study reported a close relationship between ruminal and feces metabolite profiles^29^. In addition, our recent study demonstrated that diet composition influences the ruminal and fecal microbiome in Nelore cattle^30^. Based on that, the present study hypothesized that bulls receiving distinct nutritional interventions exhibit differences in their metabolism, and ruminal fluid metabolite profiles, which may, in turn, be investigated by assessing feces metabolite profiles. Therefore, the objectives of this study were twofold: (1) to identify potential differences with respect to the ruminal fluid and fecal metabolite profiles of Nelore bulls under different nutritional interventions; and (2) to identify metabolites associated with feed efficiency (FE), water intake (WI) and methane emission (ME) phenotypes.

## Results

### Ruminal fluid and fecal sample metabolomics

For the current study, we compared the rumen fluid and feces metabolite profiles of bulls submitted to different nutritional interventions. With the initial metabolite dataset, only those metabolites with a relative standard deviation ≤ 0.15 were used for each biological sample (ruminal fluid and feces). Fifty-eight polar metabolites have previously been identified in ruminal fluid and 50 in fecal samples^29^. Furthermore, we reported here 22 and 20 fatty acids present in ruminal fluid and feces in bulls, respectively.

**Figure 1:**
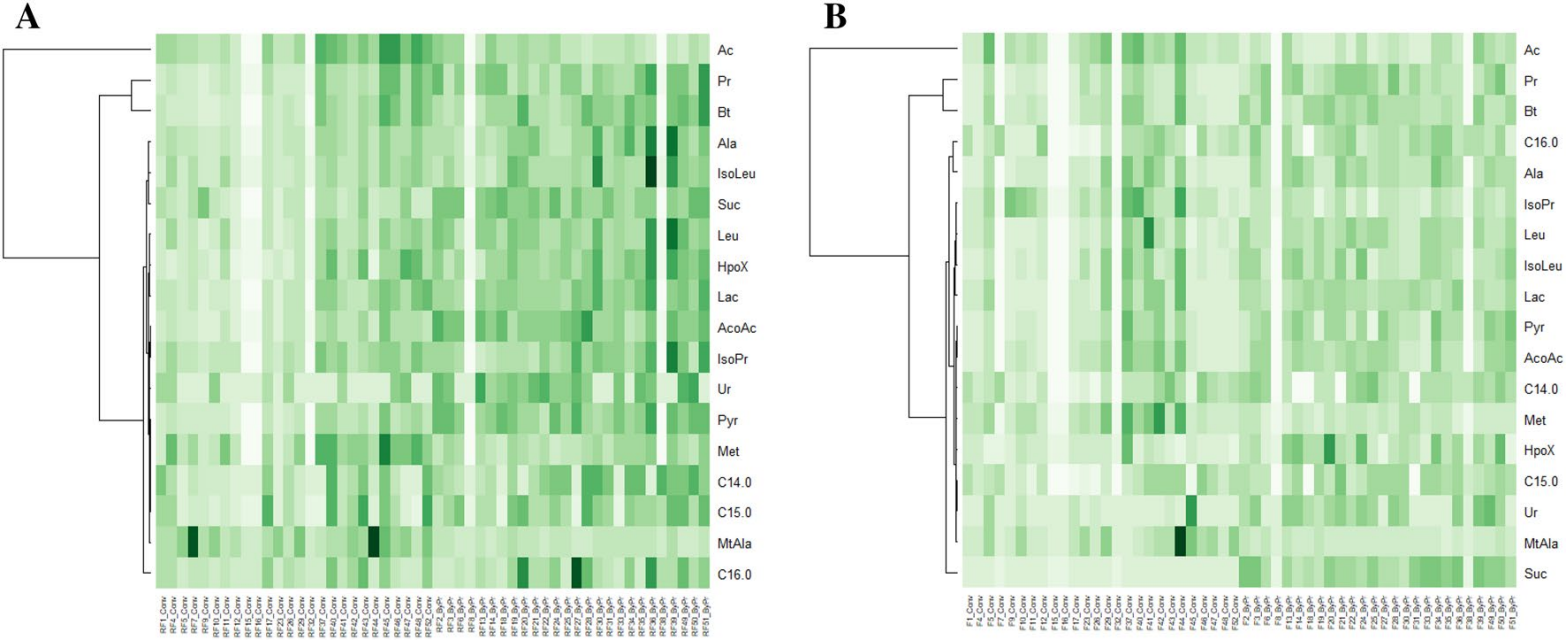
Heat maps with the corresponding dendrogram of metabolites presenting a significant difference between nutritional interventions. (**A**) Ruminal fluid metabolites were significantly different (*P* < 0.05) between the ByPr and Conv groups. (**B**) Fecal metabolites were significantly different (*P* < 0.05) between the ByPr and Conv groups. The colors vary from light green to dark green (0 to 1 data value). Ac = acetate; Pr = propionate; Bt = butyrate; Ala = alanine; IsoLeu = Isoleucine; Suc = succinate; Leu = leucine; HpoX = hypoxanthine; Lac = lactate; AcoAc = acetoacetate; IsoPr = isopropanol; Ur = uracile; Pyr = pyruvate; Met = methionine; MtAla = methylamine; C14:0 = myristic acid; C15:0 = pentadecylic acid; C16:0 = palmitic acid.

Herein, we identified 34 polar and 4apolar rumen fluid metabolites that differed significantly (*P* < 0.05) between treatments (descriptive statistics; Supplementary Table 1). Regarding fecal metabolites, 20 polar and 7apolar were significantly different (*P* < 0.05) between nutritional treatments. Furthermore, 15 polar and 3apolar metabolites were significantly different (*P* < 0.05) between the ByPr and Conv groups concerning both bio-samples (ruminal liquid and fecal samples) (Fig. 1, Supplementary Table 1). Among the metabolites, organic acids: propionate, butyrate, succinate, lactate, acetoacetate and pyruvate; amino acids: alanine, isoleucine, leucine; fatty acids: myristic (C14:0), pentadecyl (C15:0) and palmitic (C16:0); hypoxanthine and uracil were significantly higher (*P* < 0.05) in both the ruminal fluid and feces of the animals from ByPr feedlot when compared with Conv group. Acetate and methionine were significantly lower (*P* < 0.05) in both rumen fluid and feces from the ByPr group. Noteworthy, methylamine and isopropanol exhibited a significant increase in the RF while a significant decrease (*P* < 0.05) in the feces from animals of the ByPr feedlot.

**Figure 2:**
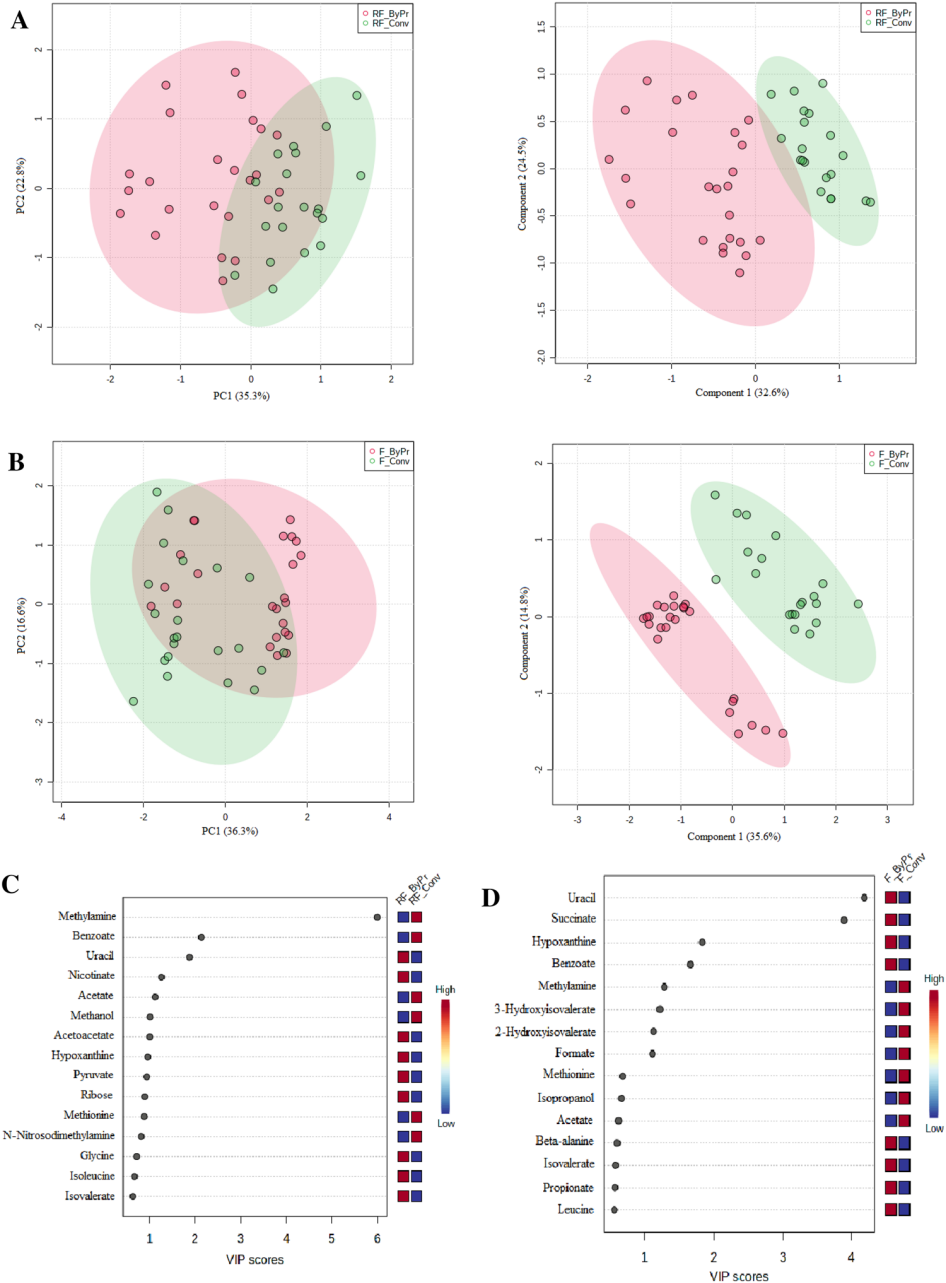
Multivariate data analysis of the metabolites: Principal Component Analysis (PCA), Partial Least Square-Discriminant Analysis (PLS-DA) and Variable importance in projection (VIP) plots. (**A**) PCA (PC1 vs. PC2) and PLS-DA (Component 1 vs. Component 2) scores plots of the Conv versus ByPr ruminal fluid metabolite profile. (**B**) PCA (PC1 vs. PC2) and PLS-DA (Component 1 vs. Component 2) scores plots metabolite profile of Conv versus ByPr feces. (**C**) VIP scores plot from PLS-DA analyses of Conv versus ByPr ruminal fluid. (**D**) VIP scores plot from PLS-DA analyses of Conv versus ByPr feces.

Our analyses demonstrated that the ruminal fluid metabolite profiles of the Conv and ByPr diets were different. A PCA and a PLS-DA (Fig. 2A) were used to visualize differences between the metabolite data. Both score plots revealed treatment differences in rumen fluid between the ByPr and Conv groups, which were well separated in the PCA [PC1 (35.3%) vs. PC2 (22.8%)] and PLS-DA [component 1 (32.6%) vs. component 2 (24.5%)]. For the first PLS-DA component, descriptive statistics from the model fitting by accuracy, estimates of the goodness of prediction (R2), and estimates of goodness prediction (Q2) were as follows: accuracy = 0.96, R2 = 0.70, and Q2 = 0.61, and accuracy = 0.98, R2 = 0.80, and Q2 = 0.67 for the first and second component, respectively.

PCA and PLS-DA plots were also constructed for fecal metabolite profiles according to nutritional treatments (Fig. 2B). The first two principal components explained 52.9% and 50.4% of the data variance for PCA and PLS-DA, respectively. The cross-validation resulted in accuracy = 0.88, Q2 = 0.61, R2 = 0.68 (component 1) and accuracy = 0.97, Q2 = 0.87, R2 = 0.91 (component 2), suggesting a clear separation of the fecal metabolites detected in the two investigated diets.

The variable importance in the projection (VIP) was performed to identify a panel of metabolites responsible for the ruminal fluid (Fig. 2C) and feces (Fig. 2D) variations between the BrPy and Conv groups. Eight metabolites of the ruminal fluid with a VIP score over 1.0 were identified (*P* < 0.05). Methylamine, acetate and methanol compounds were lower, while uracil, nicotinate, acetoacetate, hypoxanthine and pyruvate were higher in the ByPr group compared to the Conv group. A total of 4fecal metabolites that showed the greatest contribution in the discriminating PLS-DA model (VIP > 1.0) were significant (*P* < 0.05), being higher in uracil, succinate, and hypoxanthine while methylamine was lower in the ByPr diet group.

### Pathway enrichment analysis

**Figure 3:**
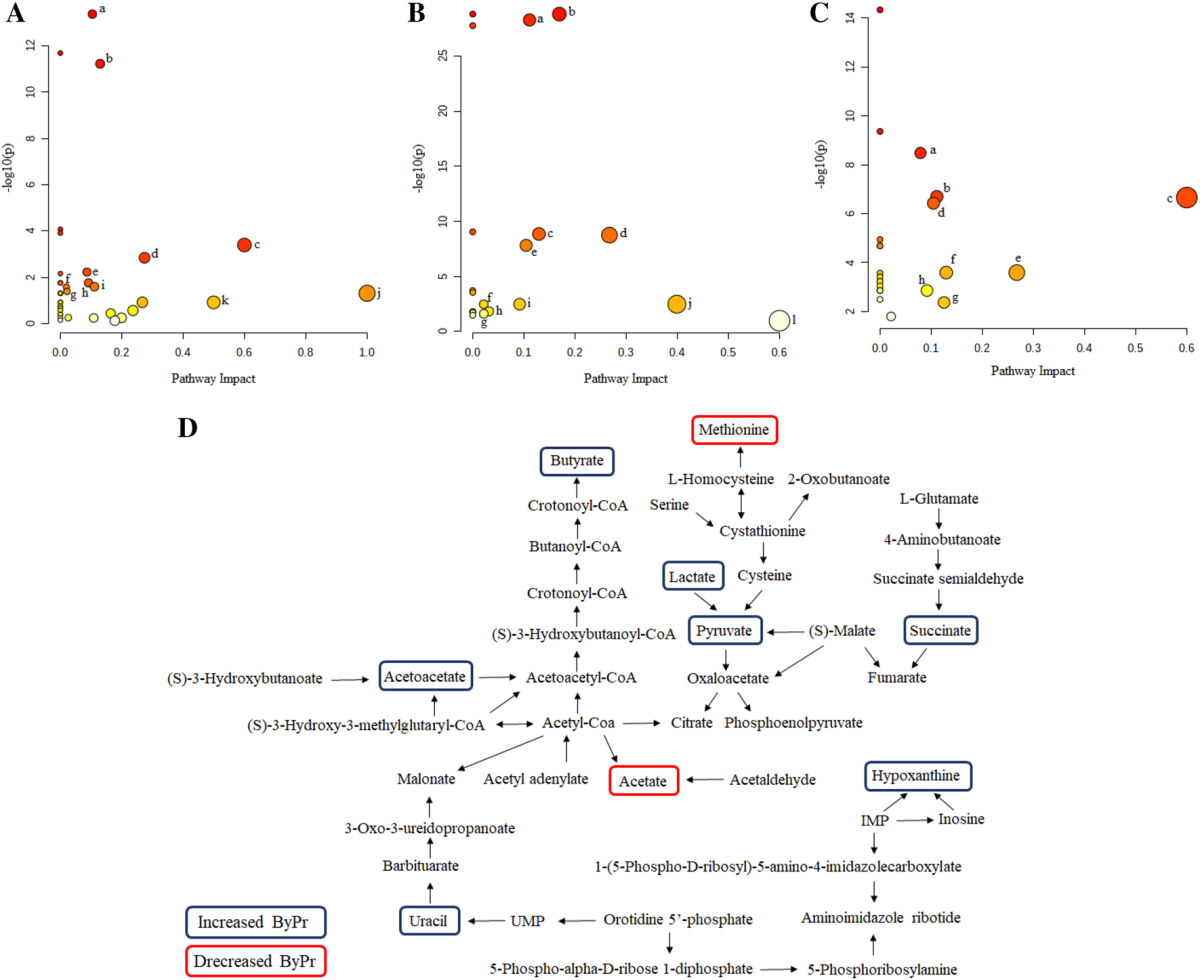
Pathway analysis using metabolites that were significantly different between the groups: (**A**) ruminal fluid, Conv versus ByPr; (**B**) feces Conv versus ByPr; (**C**) common metabolites present in rumen fluid and feces that were significantly different between diets. The x-axis displays pathway impact values from the pathway topology analysis, and the y-axis shows the *p* values from the pathway enrichment analysis. The darker the color, the more significant the pathway. The letters indicate the pathways (see Supplementary Table 2). (**D**) integrative metabolic pathway in both bio-samples (rumen fluid and feces) that were significantly different between diets (*P* < 0.05). Blue rectangle represents metabolites increased in bio-samples of the bulls that received ByPr diet, and red rectangle represent metabolites decreased in bio-samples for ByPr diet.

The significant metabolites were subjected to pathway analysis based on Metaboanalyst 5.0. The most relevant pathways that differentiated Conv ruminal fluid from ByPr ruminal fluid were cysteine and methionine metabolism; glycolysis/gluconeogenesis; synthesis and degradation of ketone bodies; arginine and proline metabolism; butanoate metabolism; d-glutamine and d-glutamate metabolism (Fig. 3A, Supplementary Table 2). The six most significantly altered pathways between feces Conv and feces ByPr were butanoate metabolism; citrate cycle (TCA cycle); glycolysis/gluconeogenesis; pyruvate metabolism; cysteine and methionine metabolism; beta-Alanine metabolism (Fig. 3B, Supplementary Table 2). In addition, enrichment analyses and integrative metabolic pathway were also performed for both bio-samples (ruminal fluid and feces) with metabolites significantly different between diets (*P* < 0.05). The results of these analyses indicate that the citrate cycle (TCA cycle); butanoate metabolism; synthesis and degradation of ketone bodies; cysteine and methionine metabolism; pyruvate metabolism; glycolysis/gluconeogenesis; pyrimidine metabolism; purine metabolism were significantly enriched *(P* < 0.05) (Fig. 3C,D, Supplementary Table 2).

### Correlation network analysis

**Figure 4:**
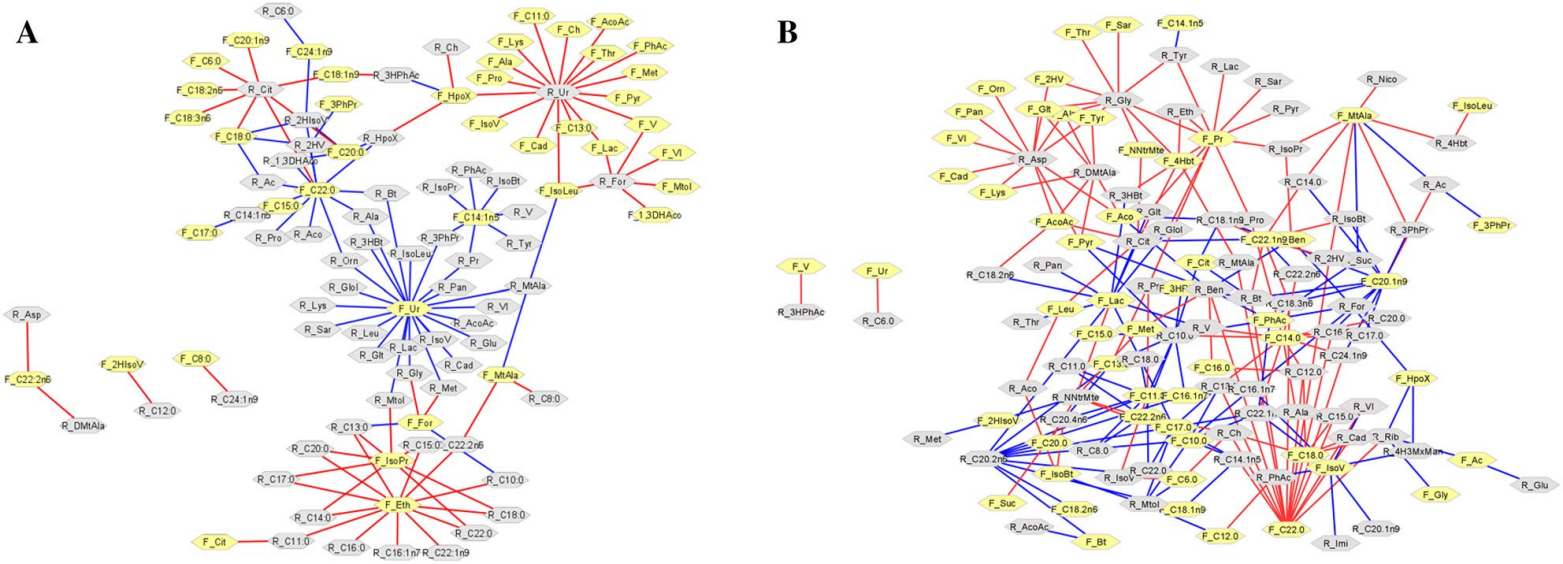
Correlation network (Spearman's correlation with *P* ≤ 0.05) carried out using the Cytoscape software. (**A**) Correlation network between ruminal and feces metabolite profiles in Nelore bulls of the Conv group (Malheiros et al. 2021). (**B**) Correlation network between ruminal and feces metabolite profiles in Nelore bulls of the ByPr group. Gray hexagons represent ruminal fluid metabolites and yellow hexagons fecal metabolites. Red lines correspond to negative correlations, whereas blue lines correspond to positive correlations between the analyzed bio-samples. The acronyms of each metabolite and correlation coefficient values are presented in Supplementary Table 3, S1, S2.

The correlation network between rumen fluid (R) and feces (F) metabolite profiles was explored. The correlations between these bio-samples from bulls of the Conv group were previously published elsewhere (Fig. 4A)^29^. In the present study, we have presented the correlations between bio-samples for the ByPr group (Fig. 4B). The ByPr group exhibited 215 significant correlations (*P* ≤ 0.05) that were used for the network construction, with 100 positive and 115 negative correlations (Supplementary Table 3, S1, S2).

The R_Asp (aspartate) was negatively correlated with fecal amino acids: lysine, alanine, glycine, tyrosine, and organic acids such as acetoacetate and pyruvate. However, F_Pyr (pyruvate) was positively correlated with R_Ben (benzoate), and negatively correlated with F_C16:0 and F_C22:0. Additionally, F_C16:0 displayed a negative correlation with R_C15:0 and R_C12:0. Ruminal fluid lauric acid was negatively correlated with F_C14:0, and negatively correlated with R_C:17:0, R_C20:0 and R_C16:0 fatty acids. Furthermore, F_C20:1n9 was negatively correlated with R_Ac (acetate) and, although positively correlated with R_Bt (butyrate). In addition, propionate identified in feces was negatively correlated to rumen fluid propionate, lactate, glycine and sarcosine. Thus, propionate, pyruvate and fatty acids are noteworthy regarding correlations between the ruminal fluid and feces of cattle fed the ByPr diet.

### Statistics and animal performance

The summary statistics of phenotypic traits for FE (ADG, MBW, FE, FCR, DMI, RFI and RADG), WI (OWI, WI and RWI) and ME (ME and RME) for the 52 Nelore bulls belonging to Conv and ByPr groups are reported in Table 1. No significant differences were identified for ADG, MBW, FE and FCR (*P* > 0.05) between the Conv and ByPr groups. However, DMI, RFI, RADG and WI traits were significantly higher (*P* < 0.05) while ME and RME traits were significantly lower (*P* < 0.05) in bulls fed ByPr compared to Conv feed.

**Table 1:** Ingredients and chemical composition of the conventional (Conv) and by-products (ByPr) diets.

	Conv	ByPr
Ingredients	%DM	%DM
Corn silage	46.58	29.98
Soybean meal	6.00	–
Corn grain	41.63	–
Protected fat	2.50	–
Peanut meal	–	7.54
Corn germ	–	35.86
Citrus pulp	–	24.01
Confinato N235 Agroceres Multimix®	2.00	2.08
Urea	1.29	0.53
Nutrients		
Dry matter, %	48.29	72.16
Crude protein, %	13.91	14.81
Neutral detergent fiber, %	30.79	30.22
Ether extract, %	5.20	6.01
Ash, %	5.20	7.37
Non-fiber carbohydrates, %	46.62	41.71
Gross energy, Mcal/kg DM	4.10	4.04
Metabolizable energy, Mcal/kg DM	2.74	2.73
Calcium, %	0.66	0.94
Phosphor, %	0.31	0.53

%DM: dry matter.

### Association analysis

Ruminal fluid and feces metabolites were associated with feed efficiency, methane emission and water intake phenotypes (Table 2). Our approach identified the aspartate and dimethylamine in ruminal fluid negatively and significantly associated (*P* < 0.05) with DMI and RFI. Choline and dimethylamine were positively associated with RADG (*P* < 0.05). FE also was positively associated with aspartate and dimethylamine (*P* < 0.05). The OWI was negatively associated (*P* < 0.05) with dimethylamine. WI was negatively associated (*P* < 0.05) with acetone, aspartate, choline and dimethylamine compounds.

**Table 2:** Adjusted means of feed efficiency, water intake, and methane emission traits at the finishing stage followed by their respective standard error.

	Conv	ByPr	*P* value
Initial body weight (BWi; kg)	404.5 ± 4.11	395.2 ± 4.31	0.6432
Final body weight (BWf; kg)	483.5 ± 6.54	484.5 ± 5.60	0.8991
Average daily gain (ADG; kg/day)	1.39 ± 0.07	1.59 ± 0.07	0.0844
Metabolic body weight (MBW; kg)	96.39 ± 0.09	95.87 ± 0.99	0.7178
Dry matter intake (DMI; kg DM/day)	7.98 ± 0.30	9.73 ± 0.31	< 0.001
Residual feed intake (RFI; kg DM/day)	− 0.96 ± 0.30	0.90 ± 0.31	< 0.001
Residual average daily gain (RADG; kg/day)	− 0.13 ± 0.05	0.12 ± 0.06	0.0029
Feed efficiency (FE; kg live weight/kg feed DMI)	0.19 ± 0.01	0.18 ± 0.01	0.5148
Feed conversion ratio (FCR; kg feed DMI/kg liveweight)	6.02 ± 0.54	6.22 ± 0.56	0.7924
Observed water intake (OWI; WI/day)	17.23 ± 1.46	21.16 ± 1.38	< 0.001
Water intake (WI; kg WI/day)	21.06 ± 0.02	21.79 ± 0.02	0.0387
Residual water intake (RWI; kg WI/day)	− 3.45 ± 0.94	1.69 ± 0.97	< 0.001
Methane emission (ME; g/day)	182.12 ± 4.7	165.23 ± 4.87	0.0107
Residual methane emission (RME; g/day)	7.53 ± 4.9	− 0.58 ± 5.16	0.2341

The fecal C22:1n9 was positively and significantly associated with MBW (*P* < 0.05). However, this metabolite was negatively and significantly associated with OWI and RWI (*P* < 0.05). The fecal metabolites C14:0 and hypoxanthine were significantly associated with DMI and RFI (*P* < 0.05). In addition, methylamine showed a positive association (*P* < 0.05) with FE.

## Discussion

### Nutrition-associated metabolome

Here we investigated the composition of ruminal and fecal metabolite profiles in Nelore bulls fed Conv and ByPr diets. Our findings show that metabolite profiles are changed in ruminal and fecal bio-samples in response to diet. Under the ByPr treatment, we identified increased propionate and butyrate concentrations in both bio-samples. Furthermore, acetate concentrations were decreased. These three volatile fatty acids (VFAs) are the main sources of metabolizable energy for ruminants^31^. Diet is a crucial factor that modulates the microbial rumen community and consequently alters VFA ratios^32–34^. VFAs can be absorbed across the ruminal wall, transported to the liver and converted into different energy sources to be used for maintenance or production^35^.

In this study, we demonstrated higher pyruvate, succinate, lactate and acetoacetate concentrations in bio-samples of the ByPr group. Pyruvate derived from glycolysis is a VFA precursor, including acetate, propionate and butyrate^36^. Acetate can be converted into butyrate in the rumen, and vice versa. However, butyrate can also be metabolized into acetoacetate^37,38^. In addition, these VFAs can be used for fat synthesis and energy supply^39^. Propionate can be produced via the succinate (pyruvate and succinate are substrates) and acrylate pathways (acrylate and lactate are substrates)^40,41^. Fumarate was observed in high concentrations in the ruminal fluid of the ByPr group. This metabolite can be reduced to succinate, which can then be used in propionate formation, and can also be used for amino acid synthesis^42^. These metabolites may be directly related to the different ingredients of nutritional interventions. The ByPr diet was composed of corn germ, which has a higher fat content. This higher-fat content can serve as a concentrated energy source and may have contributed to the stimulation of propionate production in the rumen. Additionally, the slower fermentation of lipids, compared to starch found in corn grain, could potentially contribute to a greater production of propionate. Therefore, the inclusion of corn germ in the diet may have provided an additional lipid substrate for ruminal fermentation, potentially leading to an increased production of propionate. According to Huang et al.^43^, higher propionate concentrations may aid protein synthesis, digestive enzyme action, nutrient absorption, and ruminant performance, which would be one of the reasons why animals in the ByPr group had a higher rate of gain in the present study.

Our results indicate increased levels of ruminal and fecal amino acid in the ByPr group. However, Zhang et al.^13^ reported higher ruminal amino acid concentrations, such as alanine, and proline, in response to increasing dietary concentrates (80% of concentrate). Additionally, Bica et al.^44^ observed that beef cattle fed a high concentrate diet exhibited an increased alanine and isoleucine concentration in the ruminal fluid. Alanine concentrations were altered in the ruminal fluid of cows fed high levels of barley grain (45% of diet dry matter)^45^. We found that tyrosine and valine were increased only in the ruminal fluid of the ByPr group. Li et al.^46^ reported that different diet types alter rumen tyrosine in sheep and, consequently, production performance. According to Allison and Peel^47^, valine can be generated from pyruvate and isobutyrate. Amino acids are essential in peptide and protein synthesis, as well as in metabolic pathway regulation, and microorganism growth and metabolism^43,48^. The inclusion of peanut meal in the ByPr diet, known for being a rich source of essential amino acids such as valine, is notable. Additionally, citrus pulp is utilized in animal nutrition as a source of fiber and energy. However, while it may not be widely recognized as a significant amino acid source, it could have played an indirect role in the present study by contributing to the significant levels of tyrosine and valine. Collectively, our study demonstrated that diets with distinct ingredients possibly altered the microbiota in rumen, resulting in different concentrations of ruminal and fecal amino acids in Nelore bulls.

The Conv group showed higher methylamine and lower hypoxanthine concentrations in both ruminal and fecal samples. Nitrogen metabolism produces methylamine in the rumen, which is increased when animals are fed high grain diets^34^. Bica et al.^44^ observed that methylamine and hypoxanthine are linked to a concentrated diet. Hypoxanthine is a purine derivative (adenine and guanine) resistant to rumen microbiota degradation^16^. These findings are likely due to the composition of the Conv diet, which includes soybean meal. This ingredient contains nitrogenous compounds that may contribute to the higher levels of methylamine. Additionally, the higher proportion of corn silage in the Conv diet compared to the ByPr diet, could potentially serve as a source of nucleotides and nitrogenous bases, leading to increased levels of hypoxanthine.

The fatty acids C14:0 (myristic acid), C15:0 (pentadecylic acid), and C16:0 (palmitic acid) were increased in both the ruminal and fecal samples of animals fed the ByPr diet. A 15.57% higher fat content due to the inclusion of corn germ in this diet could explain the observed higher concentrations of fatty acids in both rumen and fecal samples. Zhang et al.^49^ observed higher palmitic acid concentrations and lower myristic levels in the ruminal fluid of cows exhibiting low-yield milk production. Ruminal fatty acid concentrations indicate active lipolysis, biohydrogenation and microbial fatty acid synthesis in the rumen^50^. Further evidence of nutritional effects on ruminal and fecal metabolite profiles arose from the chemometrics analysis, in which clear group separation was observed according to the applied nutritional intervention.

### Metabolic pathway and correlations

Some rumen fluid metabolites, according to diet treatment, led to cluster formation and were directly reflected in the determination of the fecal metabolite profile. Based on a pathway analysis, we observed that synthesis and degradation of Ketone bodies, glycolysis/gluconeogenesis, butanoate metabolism, cysteine and methionine metabolism, pyrimidine metabolism and purine metabolism were the same in both ruminal and fecal samples (Conv vs. ByPr). These metabolic pathways can have significant impacts on energy processes, nutrient synthesis, and metabolic balance. In addition, our findings confirm that ruminal fluid metabolites are, thus, directly associated with fecal samples.

A correlation network was performed to compare the ruminal and fecal metabolite profiles within the ByPr group. A total of 67 ruminal metabolites were correlated with 56 feces metabolites. In a recent study by our team, 60 correlations were shown between the rumen fluid metabolites; however, only 43 fecal metabolites were correlated in the Conv group, and uracil was the main metabolite in both bio-samples^29^. Our findings suggest that correlations are mainly centered in aspartate, C20:2n6 (eicosadienoic acid), and C10:0 (capric acid) in ruminal fluid, whereas C22:0 (behenic acid), C14:0 (myristic acid), C11:0 (undecylic acid), pyruvate and lactate metabolites were central for fecal sample. Thus, the fatty acids identified in the ByPr group seem to significantly affect ruminal and fecal metabolite profiles of Nelore bulls.

### Phenotype-associated metabolome

Feed efficiency relies on the conversion of ingested feed into metabolically available nutrients^51^. This efficient conversion is an important environmental and economic factor for sustainable ruminant production^52^. In our study, we delved into the correlation between DMI and ADG traits. The analysis yielded a coefficient of − 0.12 and *P* = 0.36, indicating a non-significantly, weak negative correlation between these traits. This implies that ADG does not significantly contribute to explaining the variation in DMI, and vice versa. Furthermore, we incorporated the MBW into our model, which allowed the independence of the RFI and RADG. Consequently, we observed scenarios where animals classified as inefficient (high-RFI) had lower ADG despite having higher DMI. This situation challenges the relation the greater the DMI, the higher the ADG. Conversely, we also encountered animals with similar weight, but some bovines exhibited lower maintenance requirement, allowing it to achieve the same gain with lower DMI, classifying it as a low-RFI animals. Notably, in our study, both diets featured high energy content within the range where DMI control operates on a chemostatic basis. Consequently, the amount of feed consumed by each animal is intricately tied to its energy requirement. As a result, variation in efficiency can lead to either positive or negative relationships between DMI and ADG. Additionally, the complexity and independence of these traits underscore the importance of considering the association of each metabolite in fecal and ruminal fluid concerning each feed efficiency traits.

Thus, dietary manipulation is the most effective and convenient way to increase feed efficiency. Our findings have shown that bulls fed the Conv diet presented lower DMI and better RFI compared to ByPr. These traits were negatively associated with ruminal fluid (aspartate and dimethylamine) and fecal (C14:0) metabolites and positively associated with fecal hypoxanthine. Clemmons et al.^16^ reported that hypoxanthine in ruminal fluid was greater in low-RFI compared to high-RFI steers, however, an opposite relation was observed in feces in the present study.

In a recent study, Li et al.^53^ reported that choline affects the microbiota and is related to methane emission reduction. However, choline was not associated with methane emissions in our study. Methane production benefits the host by regulating rumen hydrogen, allowing microbial growth and promoting food digestion^54,55^. However, it results in loss of dietary host energy^56^ and is associated with greenhouse gas emissions and climate change. Thus, methane emission mitigation is paramount to reducing the environmental impact of livestock production.

Although some studies have demonstrated a positive relationship between feed efficiency and methane emissions^57^, other studies indicate non-consistency regarding this association^14,58–60^. This variable association is expected considering the cascade of physiological mechanisms related to these phenotypes. A recent review reported that, although results are somewhat controversial, the evidence seems to relate more efficient animals with higher ME^61^. In the present study, no ruminal fluid and fecal metabolites were associated with methane emissions. However, Saleem et al.^45^ found that rumen dimethylamine, methylamine, N-nitrosodimethylamine, formate, uracil, and threonine metabolites were associated with methane emissions. Bica et al.^44^ also observed ruminal metabolites as alanine, valerate, propionate, glucose, tyrosine, proline and isoleucine related to methane emissions in beef cattle fed concentrate-rich diets. However, it is important to emphasize that the relationships between metabolites and methane emissions are complex and multifactorial, influenced by interactions among diet composition, ruminal microbiome, and animal metabolic processes. Therefore, it is possible that differences in diet characteristics, breeds, animal management, or other experimental factors may have contributed to the association of these metabolites with methane emissions in the studies conducted by Saleem et al.^45^ and Bica et al.^44^.

Water intake phenotypes OWI and WI were associated with ruminal metabolites, such as acetone, aspartate, choline and dimethylamine in Nelore bulls for the first time. Fecal fatty acid C22:1n9 (erucic acid) was associated with these phenotypes as well. Water intake aids in the regulation of temperature, growth, digestion, metabolism, and excretion^62^. The studies conducted by Ahlberg et al.^63^ and Zanetti et al.^64^ demonstrated a positive relationship between WI and DMI in crossbred Angus and Nelore cattle, respectively. Furthermore, these authors inferred that adequate water consumption can help in animal growth and performance. Thus, water intake potentially impacts feed efficiency and methane emissions, and it can also influence rumen volume and fermentation. Previously, we have shown that different nutritional interventions modify the microbial community^30^. Likewise, it directly alters the rumen metabolome, leading to changes in feed efficiency, water intake and methane emission phenotypes. In addition, both the fecal and ruminal metabolomes exhibit a close relationship, regardless of diet. Ruminant digestion and feed efficiency related traits are complex. Our findings shed light on some of potential mechanisms that underlie these traits and the modulation of the ruminal and fecal metabolome; however, further investigations on the identified metabolites would provide a better understanding of their role in feed efficiency pathways, water consumption and methane emissions in beef cattle.

## Material and methods

### Production of experimental animals

All experimental procedures were conducted in accordance with animal welfare and humane slaughter guidelines and were approved by the Embrapa Livestock Science Ethics Committee on Animal Experimentation, São Carlos, São Paulo (Protocol No. 09/2016). All methods were performed in accordance with relevant guidelines and regulations. Methods are reported in the manuscript following the recommendations in the ARRIVE guidelines.

A population of 52 contemporary Nelore cattle (*Bos indicus*), progenies of 28 commercial Nelore sires, was used in an experimental feedlot. The animals were subjected to different nutritional interventions, as follows: conventional (Conv; n = 26), as described by Malheiros et al.^29^, and by-product (ByPr, n = 26). The animals assigned to the Conv and ByPr group aged 20–21 months old, with 329 ± 34.2 kg and 321.3 ± 36.3 kg of initial body weight. The bulls were allocated to collective pens containing 13 animals/pen for 105 d, of which the first 15 d were exclusively for animal adaptation, followed by the growth and finishing stages. In order to ensure ad libitum intake, the Conv and ByPr diets were offered twice a day at the finishing stage (Table 3).The pens were equipped with feeding trough (GrowSafe^®^ Ltda, Canada) and watering systems (Model AF-1000 Master, Intergado® Ltda) for automatic collection of daily feed and water intakes. Animals were weighed at the beginning and end of the feedlot period after a fasting period of 16 h, and at 28 d intervals without fasting to monitor for live weight gain. Enteric methane emissions were determined by the GreenFeed (C-lock Inc., Rapid City, SD, USA) automated system. The equipment was programmed to provide feed pellets to attract five daily visits per animal. For this study, feed efficiency parameters, residual water intake and residual methane emissions were estimated during the finishing stage.

**Table 3:** Regression coefficients of the ruminal fluid and feces metabolites associated with feed efficiency and water intake phenotypes of Nelore bulls.

	Metabolite	MBW	DMI	RFI	RADG	FE	OWI	WI	RWI
Ruminal Fluid	Acetone	− 0.14 (0.38)	− 0.66 (0.25)	− 0.68 (0.25)	2.27 (0.38)	12.61 (0.38)	− 0.001 (0.53)	− 1.04 (0.037)	0.071 (0.52 )
	Aspartate	− 0.047 (0.98)	− 6.62 (0.038)	− 6.44 (0.038)	24.70 (0.17)	177.79 (0.038)	− 0.013 (0.43)	− 6.40 (0.038)	0.067 (0.98)
	Choline	− 0.070 (0.88)	− 1.34 (0.13)	− 1.25 (0.13)	8.82 (0.079)	41.70 (0.079)	− 0.0039 (0.34)	− 1.73 (0.079)	− 0.006 (0.97)
	Dimethylamine	− 0.147 (0.81)	− 3.14 (0.085)	− 2.92 (0.098)	20.054 (0.044)	105.52 (0.035)	− 1.34 (0.095)	− 4.24 (0.035)	− 0.264 (0.58)
Feces	C14:0	0.14 (0.97)	− 4.43 (0.004)	− 4.60 (0.004)	0.84 (0.96)	73.55 (0.16)	− 0.006 (0.55)	− 2.66 (0.16)	0.042 (0.96)
	C22:1n9	0.07 (0.076)	− 0.086 (0.65)	− 0.066 (0.77)	0.242 (0.89)	4.47 (0.23)	− 0.001 (0.076)	0.03 (0.89)	− 0.06 (0.068)
	Hypoxanthine	− 0.34 (0.66)	3.86 (0.044)	3.98 (0.044)	0.83 (0.93)	− 62.18 (0.30)	0.006 (0.66)	2.22 (0.30)	0.035 (0.92)
	Methylamine	0.36 (0.48)	− 2.80 (0.19)	− 2.66 (0.19)	10.25 (0.37)	110.93 (0.058)	− 0.013 (0.19)	− 2.33 (0.24)	− 0.404 (0.38)

*Only for significantly associated metabolites (FDR adjusted *P* value ≤ 0.1), the coefficient from the linear model is given within the cell and the FDR adjusted *p* value within parentheses. *MBW* metabolic body weight, *DMI* dry matter intake, *RFI* residual feed intake, *RADG* residual average daily gain, *FE* feed efficiency, *OWI* observed water intake, *WI* water intake, *RWI* residual water intake.

### Sample collection and metabolomics analysis

After the finishing phase, fecal samples were collected from each animal, kept on ice for approximately 2 h and stored at − 80 °C until used for metabolomics assays. It is important to note that for each animal, only one ruminal liquid and one fecal sample were collected at the end of the feeding period. All animals fed with Conv and ByPr diets were sent to slaughter at 23–24 months of age and a final mean weight of 477.3 ± 41.5 and 484.7 ± 37.2 kg, respectively. At slaughter, samples of ruminal content of each animal were collected, immersed in liquid nitrogen and stored at − 80 °C for further polar and apolar metabolomics, as described by Malheiros et al.^29^.

In summary, ruminal liquid and fecal samples were solubilized in deuterium oxide phosphate buffer (0.10 M, pD = 7.4) containing 0.050% w/w of sodium 3-trimethylsilyl-2,2,3,3-d4-propionate (TMSP-d4, SigmaAldrich) and 0.02% m/v of sodium azide. Later, polar metabolites of the rumen fluid and feces were obtained by acquiring all ^1^H NMR spectra at 298 K on a 14 T Bruker Avance III spectrometer (Bruker BioSpin, Rheinstetten, Germany). The ^1^H NMR spectra were processed and metabolites were identified using the Chenomx NMR Suite Professional software package version 8.6 (Chenomx Inc., Edmonton, AB, Canada). The peaks were individually integrated and quantified using “Electronic Reference to access in vivo Concentrations 2” (ERETIC2). The apolar metabolites of rumen fluid and feces were obtained and analyzed using a GC-2014 (Shimadzu, Kyoto, Japan) gas chromatograph equipped with a flame ionization detector (FID). Quantification (mg g^−1^ of total lipids) was performed using the internal standard method using methyl ester of tricosanoic acid (C23:0).

### Feed efficiency parameters

Individual feed intake was recorded automatically and individual dry matter intake (DMI, kg/d) was calculated by multiplying the individual intake by the percentage of dry matter (DM) in the total diet. The average daily gain (ADG, kg/d) was estimated by linear regression of body weight (BW) on days in the finishing stage. The feed conversion ratio (FCR, kg/kg) was calculated as the ratio of DMI to ADG (kg/d), and the inverse of this ratio is termed feed efficiency (FE, kg/kg). Metabolic body weight (MBW, kg) was obtained with the following equation: MBW = BW^0.75^. Residual feed intake (RFI, kg/d) was calculated as the residuals from a regression of DMI in the mid-test BW^0.75^ and ADG^65^. Similarly, residual average daily gain (RADG) was calculated as the residuals from an ADG regression to mid-test BW^0.75^ and DMI^66^. The contemporary group (CG) was defined as the weighing group and slaughter group, which were considered as fixed effects by the MIXED procedure of the SAS statistical program (SAS Institute, Cary, NC, USA, 2011), according to the following equation:

$${DMI}_{i}= {\beta }_{0 }+ {\beta }_{1}\left({ADG}_{i}\right)+ {\beta }_{2 }\left({MBW}_{i}\right){+RFI}_{i}$$

where: ${DMI}_{i}$ is the dry matter intake for animal *i*; ${ADG}_{i}$ is the average daily gain of animal *i*; ${MBW}_{i}$ is the metabolic body weight of animal *i*; ${\beta }_{0}$ is the regression intercept; ${\beta }_{1}$ is the partial regression coefficient of $ADG$; ${\beta }_{2}$ is the partial regression coefficient of $MBW$, and ${RFI}_{i }$ is the RFI proposed by Koch et al.^65^ of animal *i*.

### Residual water intake

The observed water intake (OWI, kg/d) of each bovine was recorded in the finishing stage. Environmental variables were recorded by an automatic weather station of the EMBRAPA Southest Livestock, São Carlos, São Paulo. The predicted water intake (WI) was obtained with the following equation, as described by Zanetti et al.^64^:

$${WI}_{i}= {9.449+0.190 \times MBW}_{i}+ 0.271 \times {T}_{MAX}-0.259 \times HU+0.489 \times {DMI}_{i}$$

where ${WI}_{i}$ is the predicted water intake for animal *i;* ${MBW}_{i}$ is the metabolic body weight of animal *i;* T_MAX_ is the maximum temperature in °C; HU is the humidity in %; ${DMI}_{i}$ is the dry matter intake for animal *i*.

Residual water intake (RWI) was estimated by the difference between OWI and WI. The contemporary group (CG), was included in the model as a fixed effect. The MIXED procedure of the SAS statistical program (SAS Institute, Cary, NC, USA, 2011) was used, according to the following equation:

$${OWI}_{i}= {\beta }_{0 }+ {\beta }_{1}\left({WI}_{i}\right){+RWI}_{i}$$

where ${OWI}_{i}$ is observed water intake for animal *i*; ${WI}_{i}$ is the predicted water intake of animal *i*; ${\beta }_{0}$ is the regression intercept; ${\beta }_{1}$ is the partial regression coefficient of $WI$; and ${RW}_{i }$ is the residual water intake of animal *i*.

### Residual methane emission

The methane emission (ME) was observed during the finishing stage in the feedlot using the GreenFeed system (C‐lock Inc., Rapid City, SD, USA). Residual methane emission (RME) was estimated by the difference between methane emitted and individual dry matter intake (DMI, kg/d)^67^. The contemporary group (CG), was included in the model as a fixed effect. The MIXED procedure of the SAS statistical program (SAS Institute, Cary, NC, USA, 2011) was used, according to the following equation:

$${ME}_{i}= {\beta }_{0 }+ {\beta }_{1}\left({DMI}_{i}\right)+{RME}_{i}$$

where ${ME}_{i}$ is the methane emission observed for animal *i;* ${DMI}_{i}$ is the dry matter intake predicted for animal *i;* ${\beta }_{0}$ is the regression intercept; ${\beta }_{1}$ is the partial regression coefficient of $DMI$; and ${RME}_{i }$ is the residual methane emission of the animal *i* proposed by Donoghue et al.^67^.

### Metabolomics data analyses

A 0.04 ppm binning was applied to the 1H NMR data and transformed into a data matrix using the MNova software. Following identification and quantification of metabolites, as previously described, the concentrations were analyzed by MIXED procedure available in the SAS statistical program (SAS Institute, Cary, NC, USA, 2011). The statistical model included pen as fixed effect and initial and final body weights during the feedlot as covariates. Metabolite profiles were analyzed using the MetaboAnalyst 5.0 platform (http://www.metaboanalyst.ca). The data set was normalized using the Pareto scaling method. This approach means-centers the data and uses the square root of the standard deviation of each variable as a scaling factor. Multivariate analysis methods, such as Principal Component Analysis (PCA) and Partial Least Squares Discriminant Analysis (PLS-DA) were performed to identify spectral features contributing most to variation. To identify a potential panel of metabolites responsible for variations between nutritional groups, variable importance in the projection (VIP) was accessed in PLS-DA analysis. Cross-validation used leave-one-out as the procedure and accuracy as a measure to assess model validation by accuracy, the goodness of prediction (Q2) and goodness of fit (R2). Network diagrams were constructed between the determined metabolites and the impact factor of the topology analyses of metabolic pathways, graphically presented using the MetaboAnalyst 5.0 software. Finally, Spearman’s correlation analysis between ruminal and fecal metabolites was performed using Cytoscape 3.9.1 software (http://www.cytoscape.org) and plotted when *P* < 0.05.

### Metabolite-trait association

Only metabolites with a relative standard deviation > 0.15 based on the raw counts were used for association within each group^68^. Samples and metabolites were filtered out if the number of missing values was greater than 50%. After data quality control, a metabolite-trait association analysis was implemented using a linear model approach separately for ruminal fluid and feces, according to the equation:

$${y}_{ij}= {\mu }+ {T}_{i}+{P}_{j}{+ \varepsilon }_{ij}$$

where ${y}_{ij}$: is the relative concentration of each metabolite; $\mu$: is the intercept of metabolite; ${T}_{i}$ is the treatment (Conv, ByPr); ${P}_{j}$: is the trait observation for each animal (12 traits); ${\varepsilon }_{ij}$: is the random residual effect associated with each observation.

Association analysis of ruminal and fecal metabolites with the phenotypes was taken as significant when False Discovery Rate (FDR) adjusted *P* value ≤ 0.1*.*

### Supplementary Information

Supplementary Information 1.

Supplementary Information 2.

Supplementary Information 3.

## Data Availability

The data used in this study were obtained under license from Embrapa and so cannot be publicly available. Data is however available from the authors upon reasonable request, and with authorization of Embrapa.
